# Seaweed Bioactive Compounds against Pathogens and Microalgae: Potential Uses on Pharmacology and Harmful Algae Bloom Control

**DOI:** 10.3390/md16020055

**Published:** 2018-02-09

**Authors:** Soukaina El Amrani Zerrifi, Fatima El Khalloufi, Brahim Oudra, Vitor Vasconcelos

**Affiliations:** 1 Laboratory of Biology and Biotechnology of Microorganisms, Faculty of Sciences Semlalia Marrakech, Cadi Ayyad University, Av. Prince My Abdellah P.O. Box 2390, Marrakech 40000, Morocco; soukainaelamranizerrifi@gmail.com (S.E.A.Z.); elkhalloufi.f@gmail.com (F.E.K.); oudra@uca.ac.ma (B.O.); 2 Polydisciplinary Faculty of Khouribga (FPK), University Hassan 1, BP. 145, Khouribga 25000, Morocco; 3 Departament of Biology, Faculty of Sciences, University of Porto, Rua do Campo Alegre, 4169-007 Porto, Portugal; 4 CIIMAR, Interdisciplinary Centre of Marine and Environmental Research, Terminal de Cruzeiros do Porto de Leixões, Av. General Norton de Matos, s/n, 4450-208 Matosinhos, Portugal

**Keywords:** cyanobacteria, harmful algae bloom, control, microalgae, macroalgae, bioactive compounds

## Abstract

Cyanobacteria are found globally due to their adaptation to various environments. The occurrence of cyanobacterial blooms is not a new phenomenon. The bloom-forming and toxin-producing species have been a persistent nuisance all over the world over the last decades. Evidence suggests that this trend might be attributed to a complex interplay of direct and indirect anthropogenic influences. To control cyanobacterial blooms, various strategies, including physical, chemical, and biological methods have been proposed. Nevertheless, the use of those strategies is usually not effective. The isolation of natural compounds from many aquatic and terrestrial plants and seaweeds has become an alternative approach for controlling harmful algae in aquatic systems. Seaweeds have received attention from scientists because of their bioactive compounds with antibacterial, antifungal, anti-microalgae, and antioxidant properties. The undesirable effects of cyanobacteria proliferations and potential control methods are here reviewed, focusing on the use of potent bioactive compounds, isolated from seaweeds, against microalgae and cyanobacteria growth.

## 1. Introduction

Over the last two decades, there has been a growing concern about the impact of microalgae and cyanobacteria blooms due to increasing pollution and eutrophication. Harmful effects, including the development of high biomass and scums, which decrease the water quality and adversely affect the aquatic ecosystems, the aquaculture industry as well as the environmental and human health, have been reported [1]. Therefore, the control of cyanobacterial blooms is important and urgently required. Various strategies, including physical, chemical, and biological methods have been proposed for controlling or mitigating Harmful Algal Blooms (HABs). Chemical agents such us copper sulfate [2], potassium chloride [3], and endothall [4] have been used. Mechanical control involves the use of filters, pumps, and barriers [5]. Biological agents include herbivorous fishes [6], algae [7], and microorganisms [6]. However, the application of these strategies in the aquatic environment is not usually effective due to their nonselective toxicity to many aquatic organisms [8], high cost, energy expenditure, and low efficiency [9].

Recently, the isolation of natural compounds from many aquatic and terrestrial plants and seaweeds has been regarded as an environmentally friendly alternative approach for controlling harmful algae and cyanobacteria in aquatic systems [10]. These compounds include a variety of bioactive molecules such us ethyl 2-methylacetoacetate isolated from an emergent macrophyte *Phragmites communis* [11]; α-linolenic acid, oleic acid, and palmitic acid purified from *Botryococcus braunii* [12]; cyclic sulfur [13], and rutacridone epoxide [14].

Seaweeds are the most primitive group of vegetation and they have gained great importance as a promising source of bioactive compounds that can be used for drug development. Seaweeds can produce a variety of bioactive compounds, with a wide range of biological activity, including antibacterial, antifungal, antimicroalgae, antioxidant, and others [15,16,17,18]. Several live marine macroalgae (*Corallina pilulifera*, *Enteromorpha clathrata*, *Undaria pinnatifida*, *Laminaria japonica*, *Porphyra tenera*, *Ulva pertusa*, *Sargassum thunbergii*) have been found to inhibit bloom-forming microalgae such as *Cochlodinium polykrikoides*, *Skeletonema costatum*, *Heterosigma akashiwo*, and *Prorocentrum micans* [8,19,20].

## 2. Cyanobacteria

### 2.1. General Characteristics

Cyanobacteria, for a long time considered as blue-green algae on account of their ability to conduct photosynthesis, are Gram-negative bacteria. They are from a monophyletic group composed of almost 2000 species divided into 150 genera [21]. Cyanobacteria are among the oldest organisms to have appeared on our planet and are one of the most abundant and largely distributed [22]. They are present in a broad range of ecosystems such as aquatic environments (from fresh waters to hyper-saline water) and deserts [23]. They also may grow in symbiosis with algae (marine and freshwater diatoms), fungi to form lichens, with animals like protozoa, sponges or sea squirts, or with plants such as aquatic ferns, gymnosperms, and angiosperms [24]. The majority of the cyanobacteria species are aerobic photoautotrophs but some species, like *Synechocystis* sp. PCC6803 are optional heterotrophs [25]. They are responsible for about half of the earth’s oxygen atmosphere [26].

Cyanobacteria have a considerable morphological diversity. They can be solitary (unicellular), or colonial, or organized in trichomes (without sheath) or filaments (with sheath) with very varied forms (e.g., ovoid, spherical). In addition to their vegetative cells, specialized cells give them great advantages; gas vacuoles which regulate floating, the akinets which allows their conservation and dissemination, and the heterocysts which have the ability to convert dinitrogen directly (N_2_) in an available form (ammonium NH_4_^+^) through the nitrogenase. These latter types of cells are found in many kinds of cyanobacteria such as: *Microcoleus*, *Gloeothece*, *Nostoc*, *Anabaena*, *Aphanizomenon* [27,28]. Cyanobacteria can produce a variety of bioactive components, which have broad biological activity, including antibacterial, antifungal, antioxidant, and anticancer compounds [29,30,31]. According to several researchers, 40% of species of cyanobacteria are supposed to be toxigenic [32]. The toxins are classified into four large categories: neurotoxins, hepatotoxins, cytotoxins, and irritant toxins such as lipopolysaccharides [33]. Moreover, cyanobacteria also have the ability to synthesize allelopathic substances which tend to target the other competitive species directly and can induce reactions of avoidance, deteriorate their aspect, or cause their mortality [34,35].

### 2.2. Blooms of Cyanobacteria

Eutrophication is caused by an excessive load of nutritive elements which leads to changes in the aquatic environment, materialized by the proliferation of cyanobacteria blooms [36]. This situation is influenced by many factors such as temperature, pH, luminosity, and high concentrations of inorganic nutrients (nitrogen and/or phosphorus) which are often limiting elements in water bodies [36,37], as well as the stability of the water column [38].

In temperate climates, during the summer and at the beginning of the autumn, cyanobacteria blooms can form in a few days and last for one to several weeks [22], often inducing scums and leading to intense discoloration of the water bodies. The development of cyanobacteria in eutrophic mediums is supported by their reduced capacity to capture carbon dioxide (CO_2_) [39,40], the skill to use bicarbonates (HCO_3_^−^) even with raised pH, the faculty to fix and use dinitrogen (N_2_), combined with their capacity to position themselves vertically in the water column [37].

Cyanobacteria have the advantage of not being easily digested by zooplankton unlike other members of phytoplankton [41]. They secrete siderophores (hydroxamates) enabling them to capture the surrounding Fe^3+^ ions limiting the growth of potential competitors [42]. Cyanobacterial populations end up dominating the phytoplankton in eutrophic lakes. Even if blooms constitute a natural phenomenon, their frequency and their severity are increased by eutrophication, often related to anthropic activities (domestic or industrial wastewater discharge, intensive agriculture, both rich in nitrogen and phosphates). Moreover, global warming seems to act as a catalyst for cyanobacterial proliferations [43,44,45].

### 2.3. Undesirable Effects of Cyanobacteria Blooms

The harmful blooms of cyanobacteria have multiple consequences on ecosystems including the lethality of some species. Cyanobacteria massive growth can lead to two types of problems, one associated with a strong production of biomass and the other associated with the production of toxins that can result from a very low density of producing organisms [46].

The low consumption of cyanobacteria by zooplankton could disturb these trophic networks by limiting the transfers of matter and energy towards higher levels. Blooms also increase pH and water turbidity, reducing transparency and therefore light penetration. Light is then no longer available for photosynthetic activity below the surface level. In depth zones, anoxia develops and subsequently limits the growth of primary benthic producers such as macrophytes, epiphytes, and metaphyton [47]. The death of primary producers increases organic matter that causes the proliferation of decomposers (bacteria, fungi). These microorganisms mineralize organic material and use for their metabolism, dissolved oxygen which limits its access to many other organisms such as zooplankton and fish, causing significant mortalities [48], and dramatic changes in the species composition of aquatic communities [22]. Cyanobacterial blooms typically involve a considerable loss of biodiversity in the phytoplankton community [48].

The harmful blooms of cyanobacteria also generate nuisances compromising the use of water for various activities. Moreover, some cyanobacteria such as *Anabaena*, *Aphanizomenon*, *Lyngbya*, *Microcystis*, *Oscillatoria*, *Phormidium*, *Schizothrix* and *Symploca* [49], produce non-toxic volatile organic secondary metabolites, geosmin (E1, 10-dimethyl-*E*-9-decalol) and MIB (2-methyl isoborneol), which cause bad tastes and foul-smelling odors with significant economic consequences in fish farming [50,51,52]. Furthermore, more than 100 species belonging to 40 genera of cyanobacteria are able to synthesize toxins that can have harmful impacts on aquatic fauna and flora as well as the health of land animals and humans [1]. Among these genera, *Microcystis* is the most prevalent in the formation of toxic blooms, namely in Moroccan lakes [53,54,55]. Toxins are classified into four categories according to the effects they cause in mammals and vertebrates: hepatotoxins such as hepatotoxic microcystins (targeting the liver), neurotoxins (targeting the nervous system), cytotoxins and irritating toxins such as lipopolysaccharides (dermatotoxins) [33,56]. Furthermore, toxic cyanobacteria blooms in lakes may not only pose a significant threat to the drinking water supply, but may also result in significant economic losses associated with mitigation of the blooms and lake restoration [57,58]. It is estimated in the United States that the annual economic costs of eutrophication in freshwaters is over $2.2 billion [57]. In addition, the use of contaminated water by cyanotoxins in irrigation could have negative effects on the development and metabolism of seeds and plants, influencing agricultural production [59,60].

### 2.4. Methods Applied in Cyanobacterial Bloom Control

Mechanical, physical, chemical, and biological methods are used to prevent and control the blooms of cyanobacteria, the chemical ones being the most used. Copper sulfate (CuSO_4_·5H_2_O) used to be the most popular algicide. Although the treatment was usually effective by killing cyanobacteria, side effects occurred: copper is toxic to many other aquatic organisms including fish [2] and the increase in dead algal biomass led to oxygen depletion and an increase in the release of phosphorus from the sediments, resulting in the reoccurrence of the blooms.

Research showed that cyanobacteria can develop resistance to copper [61,62]. There are many other inorganic chemicals highly toxic to cyanobacteria such as potassium chloride (K^+^) [3], endothall (7-oxabicyclo(2.2.1)eptane-2,3-dicarboxylic acid) [4], and diuron (3-[3,4-dichlorophenyl]-1,1-dimethylurea) [63]. Moreover, their application to the aquatic environment is not advisable due to the nonselective toxicity to many aquatic organisms; in addition, affected populations may build up resistance to these compounds [64].

Mechanical control involves the use of filters, pumps, and barriers (curtains, floating booms) to remove or exclude algal blooms, dead fish, or other bloom-related materials from impacted waters [5]. Cyanobacterial booms can also be limited by the dilution of lake water, by lake flushing or ultrasonic radiation [65]. The object of these methods is both the augmentation of the water exchange rate and the decrease of nutrient concentration [66]. The mechanical and physical treatment of algae removal is energy intensive and tends to be of low efficiency [9]. It is applied mainly to surface scums and only a small part of the cyanobacterial population in the lake can be removed by mechanical techniques [67]. Other work however, showed that it could be effective even on a whole lake [68]. In addition, lesions caused to non-target organisms by these techniques, also limit the application in the field of such approaches in large scale.

Biological control such as biomanipulation tends to be environmentally friendly and a promising method for controlling algal blooms, being highly specific to the target organism, with no destruction of other organisms and with no direct chemical pollution. Biomanipulation involves the introduction of new grazers and competitors of cyanobacteria to control the phytoplankton development in eutrophic lakes [69]. Many organisms are used such as macrophytes and periphyton [70,71], herbivorous fishes (silver and bighead carp) [6], algae [7], and microorganisms (viruses, bacteria, fungi, and protozoa) [6]. However, the introduction of new species in an environment can have negative consequences on other species, with an imbalance of the trophic chain [72,73].

Over the last two decades, as an alternative to synthetic algicidal agents, natural compounds have been tested for controlling harmful algae in aquatic systems [10]. Research has shown that extracts and essential oils of many aquatic and terrestrial plants and seaweeds inhibit the growth of cyanobacteria. Aquatic plants, such as *Phragmites communis* [74], *Myriophyllum spicatum* [75], *Typha latifolia* and *Arundo donax* [76], *Ceratophyllum demersum* [77], *Potamogeton cristatus*, *Potamogeton maackianus*, *Potamogeton lucens*, *Vallisneria spinulosa*, *Ceratophyllum demersum*, *Hydrilla verticillata* [78] and *Sagittaria trifolia* [79], inhibit the growth of cyanobacteria. The extracts and essential oils of many terrestrial plants also show inhibitory effects against cyanobacteria, such as *Ailanthus altissima* [80], *Rosmarinus officinalis* [81], *Callicarpa americana* [82]. Moreover, several studies have demonstrated the effects of seaweeds extracts in microalgae. Sun et al. and Sun et al. [83,84], indicated that *Ulva intestinalis*, *Gracilaria lemaneiformis*, and *Ulva prolifera* inhibit the growth of various microalgae species such as *Prorocentrum micans*, *Prorocentrum donghaiense* and *Heterosigma akashiwo*.

## 3. Macroalgae

### 3.1. General Characteristics

Macroalgae, also known as seaweeds, are conspicuous and dominant features in marine ecosystems. They differ from other plants, in that algae lack roots, leafy shoots, flowers, and vascular tissues. According to differences in pigmentation, macroalgae include three different phyla: Chlorophyta, or green seaweeds are a diverse group with more than 7000 species widespread in various habitats (marine, freshwater and terrestrial ecosystems) [85]. Green algae are characterized by the dominance of two photosynthetic pigments chlorophyll a and b, chloroplasts with no outer endoplasmic reticulum, thylakoids typically in stacks of two to six, and cellulosic walls or scales. Phaeophyta, or brown seaweeds, the principal pigments in which are xanthophyll and fucoxanthin that mask chlorophyll a and c, which give them a dark shade [86]. Brown algae are distinguished by chloroplasts that have four surrounding membranes, thylakoids in stacks of three and with a richness of polysaccharides that possess importance biological activities [87]. They are exclusive to the marine habitat, under 1% of the species occur in truly freshwater habitats. Rhodophyta, or red seaweeds, the presence of two principal pigments phycoerythrin and phycocyanin, chloroplasts without external endoplasmic reticulum, unstacked thylakoids, and absence of flagella, are the principle characters of these phyla. They are prevalently marine in distribution; just roughly 3% of more than 5000 species are from fresh water [88].

Morocco due to its specific geographical position: the Mediterranean Sea to the north, the Atlantic Ocean to the west, accommodates a large bio-ecological diversity. However, the investigation of benthic kelp exhibited a particular wealth of 489 species [89] distributed between 303 species of Rhodophyceae (red algae), 99 species of Phaeophyceae (brown algae), and 87 species of Chlorophyceae (green algae). Their geographical distribution reveals the presence of 381 species (75%) on the Mediterranean coast and 323 species (64%) on the Atlantic coast, none of these algal species is endemic and only the *Gelidium sesquipedale* is currently exploited in Morocco [90].

### 3.2. Potential Use of Macroalgal Compounds

In recent years, macroalgae have gained significant importance as a new promising source of novel bioactive compounds that can be used for drug development. Seaweeds may produce a variety of bioactive compounds, which have a wide range of biological activities, including antibacterial, antifungal, antioxidant, and anti-microalgal compounds [15,16,17,18].

#### 3.2.1. Production of Antimicrobial Substances

The urgent need to find new therapeutic drugs from natural products has increased during the last decade owing to the increase of emerging multidrug-resistant microorganisms. The discovery of new bioactive substances with potent effects against resistant pathogenic and toxic microorganisms is an important aspect of the bioactive substance research today. The diversity of natural products makes it one of the most important sources of novel structures, which have been found to possess useful biological activities [91].

Generally, the antimicrobial activity of macroalgae has been extensively studied. However, the exploitation of seaweeds as a source for the discovery of new bioactive substance is still at an early stage, despite the fact that numerous novel antimicrobial compounds have been isolated over the last few years (Table 1).

**Figure 1:**
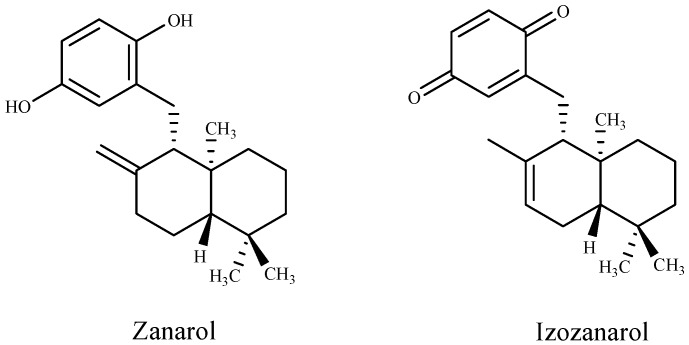
Structures of terpenoid compounds from *Dictyopteris zonarioides*.

Kamei et al. [96] found a novel antibacterial terpenoid compound, the diterpene sargafuran, from the methanolic extract of the marine brown algae *Sargassum macrocarpum*. The results of antibacterial activity show that sargafuran was bactericidal and killed *Propionibacterium acnes* by lysing bacterial cells. Also, zonarol and isozonarol sesquiterpenes (Figure 1) isolated from *Dictyopteris zonarioides* have been shown to exhibit a strong inhibitory effect against plant pathogenic fungi [95]. A few sesquiterpenoid hydroquinones occasionally incorporating halogens such as tiomanene and acetylmajapolene A and B isolated from Malaysian *Laurencia* sp. have been found to exert potent antimicrobial efficacy [93].

**Figure 2:**
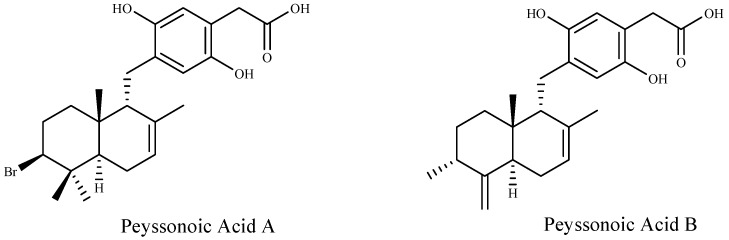
Structures of the two new sesquiterpene hydroquinones.

Furthermore, two new sesquiterpene hydroquinones, peyssonoic acids A and B (Figure 2) have been isolated from the crustose red alga *Peyssonnelia* sp. at ecologically realistic concentrations, and both compounds inhibited the growth of bacterial and fungal pathogens, *Pseudoalteromonas bacteriolytica* and *Lindra thalassiae*, from marine algae origin [92].

The antimicrobial activity may be influenced by some factors such as the habitat and the season of algal collection, different growth stages of macroalgae, experimental methods etc. Moreover the variation in antimicrobial activity may be due to the method of extraction including the solvent used in the extraction [98,99].

The potential of seaweeds as a source of active compounds against pathogenic microorganisms has been confirmed in different studies (Table 2). Taskin et al. [100] indicated that the methanolic extracts of five marine algae, *Cystoseira barbata*, *Dictyota dichotoma*, *Halopteris filicina*, *Cladostephus spongiosus* f. *verticillatus*, and *Ulva rigida* collected from the North Aegean Sea (Turkey) showed inhibition against *Staphylococcus aureus,* the most effective being *Ulva rigida* extract. Moreover, the highest inhibition activity was shown in *Enterobacter aerogenes* (34.00 ± 1.00 mm) by *Corralina officinalis* and it was followed by *Escherichia coli* and *Enterococcus faecalis*. Cortés et al. [101] found that the dichloromethane extract of *Ceramium rubrum* was active on *Yersinia ruckeri* (Gram-negative). The identification of extract composition showed that it contains fatty acids, fatty acid esters, one hydrocarbon, and phytol. In addition, they found that the antibacterial activity of the extract has a synergistic effect of its constituents because the pure compounds only showed a weak effect, which suggests a strong synergistic effect among the components. Moreover, Salvador et al. [102] screened 82 marine algae as fresh and lyophilized forms against bacterial and fungal pathogens. Of the algae 67% were active against at least one of the microorganisms tested. Among the species tested *Pseudomonas aeruginosa* was the most resistant and *Bacillus cereus* was the most sensitive. In this study, they reported that the members of the red algal order, Bonnemaisoniales were the most active. Additionally, they showed that Phaeophyceae and Rhodophyceae autumn samples exhibited the most important antimicrobial activity, while the maximum activity of chlorophyceae extracts was observed for summer samples.

According to their solubility and polarity, solvents show different antimicrobial activity. Therefore, it is necessary to select the best extraction solvents for each species of macroalgae in order to optimize extraction of the maximum chemical compounds. Methanol extracts have higher antimicrobial activity than extracts obtained with other solvents [103,104,105,106,107]. Shanmughapriya et al. [108] used fresh and dried materials of fourteen seaweeds for the extraction. They found that dried samples have less or no effects on microorganism tests in comparison to the fresh seaweed extracts. In addition, the antimicrobial principle from marine algae was found to be lipophilic. They also demonstrated that methanol extracts had higher antibacterial activity whereas ethanolic extracts had no antibacterial activity. This result was consistent with those reported by ref. [109] which showed that the methanol extraction yields had higher antimicrobial activity than ethyl acetate and hexane. They found that all seaweeds were active against at least one of the bacteria tested, on the other hand only five algal extracts showed antifungal activity. Contrary wise, Baleta et al. [110] indicated that the extraction of antimicrobials from *Sargassum oligocystum* and *Sargassum crassifolium* was solvent dependent, ethanol being the best solvent for isolation of antimicrobial compounds. Also, they revealed the presence of flavonoids, tannins, phenolics, sterols, and terpenoids which could be responsible for the observed antimicrobial property.

Radhika et al. [111] studied the antifungal activities of *Acanthophora spicifera*, *Padina tetrastomatica,* and *Caulerpascal pelliformis* against five fungal strains, namely *Aspergillus terrus*, *Aspergillus fumigatus*, *Gibberline* sp., *Alternaria* sp., and *Ganoderma* sp. The ethanol extracts showed the best antifungal activity followed by acetone and then methanol extracts. *Aspergillus fumigatus* was the most susceptible fungal species while *Ganoderma* sp. was the most resistant. However, Tüney et al. [112] investigated the antimicrobial activities of the extracts from 11 seaweed species prepared by methanol, acetone, diethyl ether, and ethanol against *Candida* sp., *Enterococcus faecalis*, *Staphylococcus aureus*, *Streptococcus epidermidis*, *Pseudomonas aeruginosa*, and *Escherichia coli*. The highest activities were obtained by the diethyl ether prepared extracts. They reported that the most active algal species was *Cystoseira mediterranea*, *Enteromorpha linza*, *Ulva rigida*, *Gracilaria gracilis*, and *Ectocarpus siliculosus* against all test organisms. Furthermore, Moorthi et al. [113] found that acetone and chloroform extracts of the *Sargassum muticum* exhibited higher antibacterial activity compared to other solvent extracts. Cox et al. [114] revealed that methanol was the better solvent for extraction of antimicrobials from Phaeophyceae; whereas acetone was good for chlorophyceae. A variety of metabolites and natural bioactive compounds groups from seaweeds, such as polysaccharides, tannins, flavonoids, phenolic acids, bromophenols, and carotenoids have been reported to be bacterial inhibitors [115,116].

Depending on their constitution and concentration, phenol compounds, chemical components of algal cells, could have an activating or inhibiting effect on microbial development [117,118]. Furthermore, seaweeds have been reported to act as inhibitors of the oxidative phosphorylation and factor cell lysis due to their ability to bind with bacterial proteins such as enzymes and those of cell membranes [116]. Wei et al. [119] reported that low molecular weight phlorotannins extracted from *Sargassum thunbergii* damaged the wall and the permeability membrane of *Vibrio parahaemolyticus* cell. Nagayama et al. [120] identified bacterial activity of phlorotannins from the brown alga *Ecklonia kurome* against 35 bacterial strains.

Marine macroalgae have been found to produce diverse bioactive compounds with antialgal activities [18,142,143] that can prevent the development of microalgae or even kill them (Table 3).

Manilal et al. [144] reported that a methanol extract of *Stoechospermum marginatum* showed significant algicidal effect and produced 90% of cell lysis of *Oscillatoria* sp. at 600 mg/L by the seventh day of treatment.

The GC-MS profile of this algal extract demonstrated the presence of diethyl phthalate as a major constituent (84.45%). Chowdhury et al. [145] investigated the toxic effect of the brown alga *Ecklonia cava* on *Cochlodinium polykrikide* and *Heterosigma akashiwo* with total growth inhibition, revealing that the maximum algicidal activity was attained after 24 h of exposition. *Ecklonia cava* potent algicidal activity against microalgae tests was maximized at a temperature of 25 °C or above. Nan et al. [146] showed that the growth of eight phytoplankton species was significantly (*p* < 0.01) suppressed in batch co-cultures with *Ulva pertusa* and the percentage of growth reduction varied between 42% and 100%. Moreover, Wang et al. [143] showed that the growth of *Heterosigma akashiwo* was strongly inhibited by using fresh tissue, dry powder or dry tissue of *Enteromorpha intestinalis*, *Ulva pertusa*, *Ulva linza*. Aqueous and methanol extracts had strong inhibitory effects on the growth of *H. akashiwo*, the effective concentration was 1.6 × 10^−12^ and 0.2 × 10^−12^ for the aqueous and the methanolic extract respectively with no apparent inhibitory effect of the other three organic solvent extracts (acetone, ether, and chloroform). Recently, Sun et al. [147] studied the effect of green alga *Ulva prolifera* on the growing of red tide microalgae and feed microalgae. The effects of *Ulva prolifera* methanolic extract partitions (F_A_, F_B_, F_C_, and F_D_) on several microalgae at the concentrations of 115.2 μg/mL F_A_ and F_B_ showed significant antialgal activity against most of the red tide microalgae tests, especially *Heterosigma akashiwo* and *Prorocentrum donghaiense*. They reported that the inhibitory activity of the fraction F_A_ on *Karenia mikimito* was lower than that on *Skeletonema costatum*; 50.3% and 100% in day 12 at 14.4 μg/m; respectively, with no biological toxicity against feed microalgae. Furthermore, after screening 27 species of seaweeds, the methanol extracts of the brown alga *Ishige sinicola* showed significant growth inhibition of more than 30% against tissue, spores, zyogote and germling of *Entermorpha prolifera*. The water extracts of two seaweeds *Codium fragile* and *Monostroma nitidum* showed significant growth inhibition of more than 40% against tissue of *Entermorpha prolifera*, and only one seaweed *Porphyra yezoensis* showed significant inhibition of more than 30% against zygote of *Entermorpha prolifera* [148].

**Figure 3:**
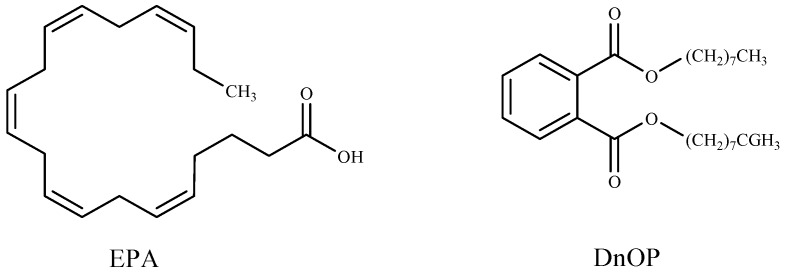
Algicidal substances isolated from *Corallina pilulifera*.

Researchers have described methods of controlling cyanobacteria harmful blooms by using algicidal compounds extracted from seaweeds, such as octadeca-6*Z*,9*Z*,12*Z*,15*Z*-tetraenoic acid (ODTA) isolated from the brown alga *Cladosiphon okamuranus* [160]; α-linolenic acid, oleic acid, and palmitic acid isolated from green alga *Botryococcus braunii* [12]; hexadeca-4,7,10,13-tetraenoic acid (HDTA), octadeca-6,9,12,15-tetraenoic acid (ODTA), and α linolenic acid isolated from the green alga *Ulva fasciata* [142]; (6*E*,9*E*,12*E*)-(2-acetoxy-β-d-glucose)-octadecatrienoic acid ester separated from green alga *Ulva intestinalis* [83]; gossonorol, 7,10-epoxy-ar-bisabol-11-ol, glycerol monopalmitate, stigmasterol, 15-hydroxymethyl-2,6,10,18,22,26,30-heptamethyl-14-methylene-17-hentriacontene, 4-hydroxyphenethyl alcohol, and margaric acid were obtained from the ethanolic extract of the red alga *Gracilaria lemaneiformis* [84]; 5,8,11,14,17-eicosapentaenoic acid (EPA) and di-n-octylphthalate (DnOP) (Figure 3) purified from the methanol extract of the red alga *Corallina pilulifera* [157].

**Figure 4:**
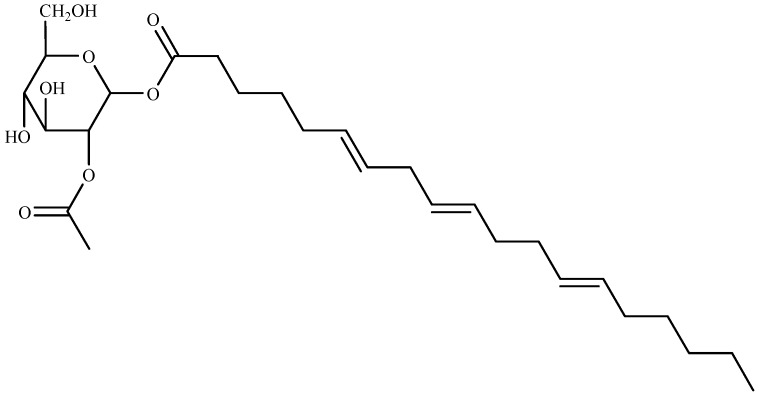
Structure of compound II isolated from *Ulva intestinalis*.

Recently, three algicidal compounds in the ethyl acetate (EtOAc) extracts were successfully isolated from green algae *Ulva intestinalis* as 15-ethoxy-(6*Z*,9*Z*,12*Z*)-hexadecatrienoic acid (I), (6*E*,9*E*,12*E*)-(2-acetoxy-β-d-glucose)-octadecatrienoic acid ester (II), and hexadecanoic acid (III). Compound I and III showed moderate algicidal activity. Whereas compound II (Figure 4) displayed the most potent algicidal activity with IC_50_ values of 4.9 and 14.1 μg/mL for *Heterosigma akashiwo and Prorocentrum micans*, respectively [83].

**Figure 5:**
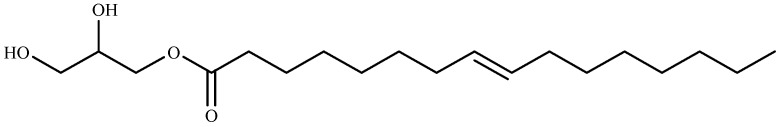
Structure of 9-hexadecenoic acid, 2,3-dihydroxypropyl ester isolated from *Ulva prolifera*.

Ten compounds were identified for the first time from green algae *Ulva prolifera* as three glycoglycerolipids: 1-*o*-octadecanoic acid-3-*o*-β-d-galactopyranosyl glycerol (2), 1-*o*-palmitoyl-3-*o*-β-d-galactopyranosyl glycerol (4), and 1-*o*-palmitoyl-2-Ooleoyl-3-*o*-β-d-galactopyranosyl glycerol (5); two monoglycerides: glycerol monopalmitate (1), 9-hexadecenoic acid, 2,3-dihydroxypropyl ester (3); two terpenoids: loliolide (6), and lsololiolide (7); one lipid-soluble pigments: zeaxanthin (8); one sterol: cholest-5-en-3-ol (9); and one alkaloid: pyrrolopiperazine-2,5-dione (10). Their algicidal activity reveal that compounds **3**, **6**, and **7** showed the stronger activity. The results also prove that compound **3** (9-hexadecenoic acid, 2,3-dihydroxypropyl ester) (Figure 5), was isolated for the first time from marine macroalgae [147].

#### 3.2.2. Antioxidant Activity

Among all the compounds contained in macroalgae, antioxidants are the most abundant. They can be classified into two groups, exogenous (vitamin C, vitamin E, and polyphenols) and endogenous antioxidants (enzymes and proteins) [161]. Seaweeds, like all photosynthesizing plants, are exposed to free radical and strong oxidizing agents due to a combination of high light and high oxygen concentration [162,163]. However, the absence of structural damage in the cells of macroalgae and their stability to oxidation during storage, suggests that these cells have protective antioxidative mechanisms and compounds [164,165].

Several studies have investigated the antioxidant activity of natural products in seaweeds. Chang and Teo [161] studied the antioxidant activity of *Eucheuma cottonii* extract by 2,2-diphenyl-1-picrylhydrazyl (DPPH) scavenging method. The result showed that the total phenolic content value for the seaweed extract was 3.40 ± 0.013 mg GAE/g, and the IC_50_ of *E. cottonii* crude extract on DPPH was 38.82 ± 0.99 mg/mL. The antioxidant activity of extracts of 48 species of seaweed collected from the coasts of Yucatan and Quintana Roo (Mexico) was evaluated by DPPH scavenging method. All species exhibited a DPPH radical scavenging activity, and *Avrainvillea longicaulis* demonstrated the largest antioxidant potential with a very low oxidation index EC_50_ (1.44 ± 0.01 mg/L) with high phenolic content (3.36 ± 0.05% dry wt.), while the lowest antioxidant activity was observed in *Enteromorpha intestinalis* (43.23 ± 0.28) [166]. The in vitro antioxidant activities of methanol extracts of five selected species (*Codium tomentosum*, *Enteromorpha linza*, *Gelidium sesquipedale*, *Cystoseira spicata,* and *Padina pavonica*) of Libyan algae were evaluated by Alghazeer et al. [139]. They found that the maximum antioxidant activity was exhibited significantly by the methanol extract of *Cystoseira spicata* 199.38 ± 12.73 (199.38 mg of ascorbic acid/g of seaweed dry weight) with a significant high amount of phenolics, flavonoids, and condensed tannins compared with the other extracts whereas, the extract of the green algae *Enteromorpha linza* exhibited the lowest antioxidant activity (144.05 mg of ascorbic acid/g of seaweed dry weight). Lee et al. [167] studied the in vivo antioxidant activities of fucosterol isolated from the marine algae *Pelvetia siliquosa*. The results showed that fucosterol produced a significant increase of free radical scavenging enzyme activities such as hepatic cytosolic superoxide dismutase (SOD), catalase, and glutathione peroxide (GSH-px) activities by 33.89%, 21.56%, and 39.24%, respectively.

**Figure 6:**
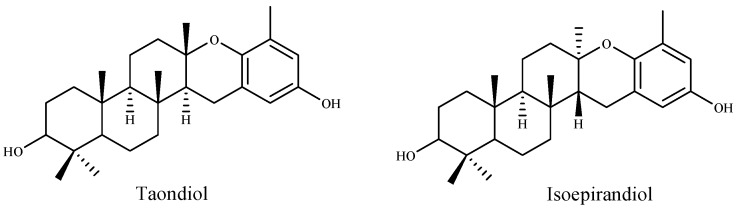
Antioxidant metabolites of the brown alga *Taonia atomaria*.

Many researchers have indicated a relation between total phenolic and flavonoid content and high antioxidant activity. Farasat et al., Chai et al. and Alghazeer et al. [168,169,170] reported a positive correlation between antioxidation capacity and the total phenolic and flavonoid contents. Pinteus et al. [171] attributed the strong antioxidant activity to the high phenolic content. They also suggested that high antioxidant activity is not directly linked to a high cytoprotective potential. Contrariwise, Lim et al. and Mamelona et al. [165,172] demonstrated that the antioxidant capacity is not directly correlated with the total phenolic contents. Also, Cho et al. [173] suggested that the antioxidant activity of the extracts from the green algae *Enteromorpha prolifera* was related to the chlorophyll compound pheophorbide, and not to total phenolic contents. According to ref. [137] the free radical scavenging activity on DPPH was found to be increased with the increase of concentration of methanolic extract of *Ulva lactuca*. In this study, the IC_50_ value was lower (81.36 µg/mL) compared to other reported values [161]. Recently, Raja et al. [174] suggested that the antioxidant potential of *Eisenia arborea* was the most effective followed by *Ulva lactuca* and *Codium fragile*. The methanolic extracts were found to contain high phenolic and flavonoid contents with higher antioxidant activities compared to their aqueous extract. Nahas et al. [175] tested the radical scavenging activity (RSA) of thirteen algae from the Aegean Sea by using the DPPH test and chemi-luminescence (CL). The results indicated that the extracts of the brown alga *Taonia atomaria* exhibited the best RSA in comparison to the other algae extracts. Moreover, they suggested that two metabolites, taondiol and isoepitaondiol (Figure 6), were responsible for the extract antioxidant activity.

## 4. Conclusions

Enhanced growth of aquatic vegetation or phytoplankton and algal blooms disrupts normal functioning of aquatic ecosystems all over the world. When toxic microalgae and cyanobacteria are involved in these eutrophication consequences, a variety of ecological, economical, and sanitary health problems could arise. Most of the recent studies on the control of the HABs have focused on the use of chemical, physical, and biological treatment agents but these processes show serious environmental consequences. Among the biological agents, a variety of extracts from aquatic and terrestrial plants, which contain many bioactive compounds, with a wide range of applications and biocides activities have been experimented.

With respect to bioactive compounds extracted from seaweeds, most of them have been applied for their biocidal (anti-fungi, anti-bacteria) and pharmaceutical activities. However, very few reports have focused on their algicide and anti-cyanobacterial activities. In order to explore macroalgae as an alternative and an available natural source of bioactive compounds, we recommend that works could be oriented on the research of new natural products extracted from seaweeds. Seaweed extracts show interesting potential against many harmful microalgae and cyanobacteria species but not much is yet known, namely the structure and mechanisms of action of the effective substances. These substances should be tested for their biocide activities against micro-algae growth in general and particularly against cyanobacteria growth. Research on novel biomolecules is needed in order to better control the phytoplankton excessive growth in a sustainable way, and to maintain the ecological equilibrium and the stability of the aquatic ecosystems.

## Figures and Tables

**Table 1:** Novel antimicrobial compounds isolated from seaweeds.

Compound	Source	Reference
Peyssonoic acid A and B	*Peyssonnelia* sp.	[92]
Tiomanene Acetylmajapolene (A and B)	*Laurencia* sp.	[93]
3-Dibromobenzaldehyde-4,5-disulfate potassium salt 5-Bromo-3,4-dihydroxybenzaldehyde	*Polysiphonia lanora*	[94]
Zonarol and isozonarol sesquiterpenes	*Dictyopteris zonarioides*	[95]
Diterpene sargafuran	*Sargassum macrocarpum*	[96]
10-Hydroxy kahukuene B	*Laurencia mariannensis*	[97]

**Table 2:** Antibacterial and antifungal activity of different solvent extracts from seaweeds.

Solvents	Seaweed	Target Organisms	Reference
Acetone	*Turbinaria conoides*	*Fusarium oxysporum*	[121]
	*Ulva lactuca*	*Escherichia coli*, *Staphylococcus aureus*, *Bacillus mycoides*, *Bacillus subtilis*, *Klebsiella pneumoniae*, *Aspergillus flavus*, *Aspergillus fumigatus*, *penicillium purpurescens*, *Candida albicans* and *Penicillium verrucosum*	[122]
Acetone, Chloroform	*Sargassum muticum*	*Micrococcus* sp., *Staphylococcus aureus* (*methicillinresistance*), *Salmonella paratyphi B*, *Staphylococcus epidermis*, *Enterobacter aerogenus*, *Shigella fleschneri*, *Proteus vulgaris*, *Staphylococcus aureus Salmonella typhymurium*	[113]
Acetone, Ethyl, acetate, Hexane	*Sargassum wightii*, *Chaetomorpha linum* and *Padina gymnospora*	*Erwinia amylovora*, *Enterobacter aerogenes*, *Proteus vulgaris*, *Escherichia coli*, *Staphylococcus aureus*, *Bacillus subtilis Enterococcus faecalis*	[98]
Acetone, Ethyl acetate, Hexane, Methanol	*Chaetomorpha linum*	*Pseudomonas aeruginosa Bacillus subtilis*	[123]
Acetone, Methanol	*Sargassum platycarpum*, *Sargassum latifolium*	*Escherichia coli*, *Salmonella* sp., *Staphylococcus xylosus*, *Staphylococcus aureus*, *Bacillus subtilis*, *Enterococcus faecalis Candida albicans*	[124]
Benzene, Diethyl ether, Ethyl acetate, Hexane	*Chlorococcum humicola*	*Escherichia coli*, *Pseudomonas* *aeruginosa*, *Salmonella typhimurium*, *Klebsiella pneumoniae*, *Vibreo cholerae*, *Staphylococcus aureus*, *Bacillus subtilis*, *Candida albicans*, *Aspergillus niger* and *Aspergillus flavus*	[125]
Chloroform, Ethanol	*Ulva reticulata*, *Caulerpa occidentalis*, *Cladophora socialis*, *Dictyota ciliolata*, and *Gracilaria dendroides*	*Escherichia coli*, *Pseudomonas aeruginosa*, *Stapylococcus aureus Enterococcus faecalis*	[126]
Chloroform, Hexane, Ethyl acetate, Methanol	*Ulva lactuca*, *Sargassum polyceratium*, *Caulerpa racemosa*	*Bacillus subtilis*, *Micrococcus luteus*, *Staphylococcus aureus*, *bacteria Escherichia coli* and *Klebsiella pneumoniae*	[127]
Chloroform, Hexane, Ethyl acetate, Methanol	*Jania adhaerens*, *Padina gymnospora*	*Bacillus subtilis* and *Micrococcus luteus*	[127]
Diethyl ether, Methanol, Ethanol	*Ceramium rubrum*, *Sargassum vulgare*, *Sargassum fusiforme* and *Padina pavonia*	*Pseudomonas aeruginosa*, *Shigellaflexneri*, and *Klebsiella pneumoniae*	[128]
Ethanol	*Stypocaulon scoparium* and *Halopitys Incurvus*	*Staphylococcus aureus*, *Bacillus subtilis*, *Escherichia coli*, *pseudomonas aeruginosa*, *Fusarium oxysporum f.* sp. *Albedinis* and *Penicillium* sp.	[129]
	*Asparagopsis taxiformis*	*Aspergillus fumigatus*, *Aspergillus terreus* and *Aspergillus flavus*	[130]
Ethyl acetate	*Eisenia bicyclis*	*Propionibacter iumacnes*, *Staphylococcus aureus*, *Staphylococcus epidermidis* and *Pseudomonas aeruginosa*	[131]
Methanol	*Ulva lactuca*, *Sargassum wightii* and *Gracilaria edulis*	*Bacillus cereus*, *Streptococcus faecali*, *Staphylococcus aureus*, *E-coli*, *Pseudomonas aeruginosa*, *Salmonella typhi* and *Vibreo cholerae*	[132]
	*Ulva rigida* and *Ulva intestinalis*	*Esherichia coli*, *Streptococcus pyogenes*, *Staphylococcus epidermidis*, *Candida albicans* and *Aspergillus niger*	[133]
	*Ulva lactuca*, *Ulva fasciata Enteromorpha compressa*, *Pterocladia capillacea*, *Hypnea musciformis* and *Padinapavonica*	*Fusarium solani*, *Fusarium oxysporum*, *Tricodermahamatum*, *Aspergillus flavipes* and *Candida albicans*	[134]
	*Ulva lactuca*	*Staphylococcus aureus* and *Pseudomonas aeruginosa*	[135]
	*Sargassum wightii*	*Staphylococcus aureus.*, *Klebsiella pneumonia*, *Proteus mirabilis*, *Escherichia coli* and *Proteus valgaris*	[136]
	*Ulva lactuca*	*Bacillus subtilis*, *Corynebacterium diphtheria*, *Staphylococcus aureus*, *Escherichia coli*, *Pseudomonas aeruginosa*, *Salmonella paratyphi*, *Aspergillus niger Aspergillus fumigatus*	[137]
	*Turbinaria ornata*, *Padina tetrastromatica*	*Micrococcus luteus* and *Bacillus subtilis*	[138]
Methanol, Water	*Cytoseira crinite*	*Taphylococcus aureus*, *Bacillus subtilis*, *Bacillus* spp., *Staphylococcus epidermidis Escherichia coli and Salmonella typhi*, *Klebsiella* spp., *and Pseudomonas aeruginosa*	[139]
Polysaccharides	*Corallina*	*Staphylococcus epidermidis*, *Staphylococcus aureus*, *Enterococcus feacalis Escherichia coli* and *Pseudomonas aeruginosa*	[140]
Toluene	*Gracilaria crassa*, *Gracilaria debilis*, and *Gracilaria corticata*	*Escherichia coli*, *Shigella* sp., *Staphylococcus aureus*, *Vibriocholerae*, *Proteus* sp., *Bacillus subtilis* and *Pseudomonas fluroscens*	[141]

**Table 3:** Anti-microalgal activity of different extracts from seaweeds.

Macroalgae	Fraction Used or Solvent	Target Species	Effects	Reference
*Enteromorpha intestinalis*	Ethanol extract Fresh tissue	*Prorocentrum micans*	Fresh tissue and ethanol extracts significantly inhibited the growth of *P. micans.*	[149]
*Sargassum thunbergii*	Methanol extract	*Heterosigma akashiwo* *Skeletonema costatum* *Prorocentrum micans*	Stronger inhibitory effects on the growths of red tide microalgae tests.	[20]
*Enteromorpha clathrata*, *Undaria pinnatifida* *Laminaria japonica* *Porphyra tenera* Ulva pertusa	Seawater extracts	*Skeletonema costatum*	The macroalgal extracts of *P. tenera*, *E. clathrata*, and *U. pertusa* showed strong growth inhibition on *S. costatum.*	[19]
*Gracilaria lemaneiformis* *Ulva pertusa*	Fresh thalli Water-soluble extract Dry powder	*Heterosigma akashiwo*	Algicidal effects of both macroalgae on *H. akashiwo*, cells were entirely killed.	[150]
*Gracilaria tenuistipitata*	Dry powder	*Prorocentrum micans*	Inhibitory effect on the photosynthesis of *P. micans*.	[151]
*Ulva lactuca*	Dry powder Fresh thalli Extracts	*Aureococcus anophagefferens* *Cochlodinium polykrikoides* *Pseudo-Nitzschiamultiseries* *Prorocentrum minimum* *Karlodinium veneficum* *Chattonella marina* *Karenia brevis*	The fresh thalli and dry powder strongly inhibited the growth of all seven HAB species with advantage of dry powder. The extracts of *U. lactuca* exhibited dramatic allelopathic effect on the HAB species.	[152]
*Sargassum thunbergii* *Corallina pilulifera* *Ulva pertusa*	Aqueous extracts Fresh tissue Dry powder	*Heterosigma akashiwo* *Alexandrium tamarense*	The growth of the two microalgae was strongly inhibited	[153]
*Asparagopsis taxiformis*	Methanol extract	*Trichodesmium* sp.	Total inhibition of *Trichodesmium* sp. growth	[154]
*Hypnea musciformis*	Methanol extract	*Isochrysis galbana* *Chlorella salina*	Enhancement growing of both microalgae even at low concentration	[155]
*Gracilaria lemaneiformis*	Ethanol extract	*Prorocentrum donghaiense* *Skeletonem acostatum* *Heterosigma akashiwo* *Amphidinium carterae* *Phaeocystis globa* *Karenia mikimitoi*	Inhibitory effect on the growth of all microalgae	[84]
*Ulva pertusa*	Methanol extract	Red tide microalgae Feed microalgae	The methanolic extract showed antialgal activity against red tide, with no growth inhibition for feed microalgae.	[156]
*Corallina pilulifera*	Methanol extract	Red tide microalgae Feed microalgae	Stronger inhibitory effects on the growths of red tide microalgae, with no growth inhibition for feed microalgae.	[157]
*Ulva intestinalis*	Ethanol extract Fresh tissue Dry powder	*Heterosigma akashiwo* *Prorocentrum micans*	The fresh tissue, dry powder and extract, all exhibited obvious algicidal effects on red tide microalgae.	[83]
37 species	Methanol extract Water extract	*Heterosigma akashiwo*	The green alga *Ulva fasciata* showed the strongest algicidal activity among the 37 seaweeds tested	[142]
*Ulva lactuca* *Ulvafasciata*	Ethanol extract	*Chlorella vulgaris*	Stimulation of growth and progressive increase of *Chlorella vulgaris* biomass	[158]
*Ulva pertusa* *Ulva prolifera*	Extracts (acetone, ether chloroform, methanol) Fresh tissue Dry powder	*Prorocentrum donghaiense*	Stronger inhibitory growing effects by fresh tissue and dry powder of both seaweeds. Methanol extracts of the macroalgae were found to strongly inhibit the growth of *P. donghaiense*.	[159]
*Ecklonia kurome*	Phlorotannins extract	*Cochlodinium polykrikoides* *Chattonella antiqua* *Kareniam ikimotoi*	Destruction of 99% cells of ride tide microalgae, with no mortality observed among other organisms such us: *Pagrus major*, tiger puffer Fugu rubripes or larval blue crab *Portunustrituberculatus*.	[120]
*Ulva pertusa*	Fresh tissue	*Heterosigma akashiwo*, *Skeletonema costatum*, *Tetraselmis subcordiformis*, *Nitzschia closterium*, *Chaetoceros gracile*, *Chroomonas placoidea*, *Isochrysis galbana*, *Alexandrium tamarense*	Algicidal interaction between green alga *Ulva pertusa* and all phytoplankton species	[146]
